# Bleeding at the Cord

**Published:** 1877-02-20

**Authors:** A. Petit


### BLEEDING AT THE CORD.

BY DR. A. PETIT.

*Case* 1. Attended a lady who, after a natural labor, was delivered of a large, healthy looking male child. There was nothing abnormal in the appearance of the cord, which I ligated as usual. About three hours after leaving the house I was called back, and found that there had been a severe hemorrhage from the cord. There was no bleeding when I arrived, but though the ligature seemed sufficiently tight, I placed another behind it. There was no more bleeding at that time, but on two occasions after the dropping of the cord there were slight hemorrhages from the umbilicus, for which I used the topical application of the solution of perchloride of iron. The child survived and did well.

*Case* 2. Was called to see an infant three days old. It presented a good appearance as to health, but had just had a severe hemorrhage from the base of the undetached cord; there had been no bleeding from the distal end of the cord. I wrapped the base of the cord with linen saturated with the solution of perchloride of iron, and ordered one drop of the muriated tincture of iron and five of Squibb's ergot every two hours. There was no return of the hemorrhage then or thereafter.

*Case* 3. Was called to see a negro child eight or ten days old, which was having a severe hemorrhage from the umbilicus; tried the topical application of perchloride of iron without success, then transfixed the unbilicus with two pins and wrapped them tightly with silk; this controlled the hemorrhage for a while, but the child ultimately bled to death.

*Case* 4. Was called during the night to see a child about eighteen days old, which presented a very anæmic appearance. Father stated that at birth it seemed healthy and promising, but that on two occasions after the dropping of the cord it bled profusely from the umbilicus—afterwards from the skin on the right side of the chest, and then from the right and left temple. After this a large blood tumor made its appearance on the left wrist. The seat of hemorrhage on the right temple was covered by a little desicated blood about the size of a pin's head, and the seat of hemorrhage from the left temple presented a little rosy spot about the same size. There was no apparent solution of continuity of the skin. The left wrist presented a large blood tumor, the skin covering of which was dark and seemed about to sphacelate. There had also been some hemorrhage from the throat, which, on being examined, presented a dark, apparently hemorrhagic tumor on the edge and above the right soft palate. The father now told me that his family physician was attending the child, but that having tried unsuccessfully to get him to open the tumor on the wrist, he had sent for me. I told him the child would most probably die any how, but that the use of any cutting instrument would insure and hasten its death. He told me afterwards, that on the next day his physician yielded to his importunities and lanced the tumor, after which the child bled to death. I think the frequent administration of small doses of iron and ergot, and the application of a compress of carbolized glycerine to the tumor would have been a treatment worthy of trial. I made inquiries as to the health of the parents. They both looked well, and I could find no reason to attribute the bad nutrition of this child to constitutional taint. They gave healthy histories, and had had several other children who enjoyed invariable good health.—*N. O. Med. Jour.*

